# Factors influencing contraceptive use and non-use among women of advanced reproductive age in Nigeria

**DOI:** 10.1186/s41043-016-0077-6

**Published:** 2017-01-07

**Authors:** Bola Lukman Solanke

**Affiliations:** 1 Department of Demography and Social Statistics, Obafemi Awolowo University, Ile-Ife, Nigeria; 2 Demography and Population Studies Programme, University of the Witwatersrand, Ile-Ife, South Africa

**Keywords:** Modern contraceptive, Advanced reproductive age, Contraceptive use, Contraceptive non-use, Traditional method, Women, Nigeria

## Abstract

**Background:**

Factors influencing contraceptive use and non-use among women of advanced reproductive age have been insufficiently researched in Nigeria. This study examines factors influencing contraceptive use and non-use among women of advanced reproductive age in Nigeria.

**Methods:**

Secondary data were pooled and extracted from 2008 and 2013 Nigeria Demographic and Health Surveys (NDHS). The weighted sample size was 14,450 women of advanced reproductive age. The dependent variable was current contraceptive use. The explanatory variables were selected socio-demographic characteristics and three control variables. Analyses were performed using Stata version 12. Multinomial logistic regression was applied in four models.

**Results:**

Majority of the respondents are not using any method of contraceptive; the expected risk of using modern contraceptive relative to traditional method reduces by a factor of 0.676 for multiparous women (rrr = 0.676; CI: 0.464–0.985); the expected risk of using modern contraceptive relative to traditional method reduces by a factor of 0.611 for women who want more children (rrr = 0.611; CI: 0.493–0.757); the relative risk for using modern contraceptive relative to traditional method increases by a factor of 1.637 as maternal education reaches secondary education (rrr = 1.637; CI: 1.173–2.285); the relative risk for using modern contraceptive relative to traditional method increases by a factor of 1.726 for women in richest households (rrr = 1.726; CI: 1.038–2.871); and the expected risk of using modern contraceptive relative to traditional method increases by a factor of 1.250 for southern women (rrr = 1.250; CI: 1.200–1.818).

**Conclusions:**

Socio-demographic characteristics exert more influence on non-use than modern contraceptive use. The scope, content and coverage of existing BCC messages should be extended to cover the contraceptive needs and challenges of women of advanced reproductive age in the country.

## Background

Contraceptive use particularly modern contraceptive use remains prominent in demographic and health literature because of its numerous health benefits to women and families such as preventing unintended pregnancies, promoting healthy birth spacing, reducing lifetime risk of maternal deaths, and enhancing attainment of development goals [1–4]. In addition, contraceptive use remains a dominant population and health issue because of its important role in the demographic transitions in different countries with varying degrees of demographic situations [5]. Large numbers of studies across the world have examined individual, institutional and community determinants of contraceptive use among different groups of women [6–12]. However, there is a paucity of studies focusing on the dynamics of contraceptive use and non-use among women in advanced reproductive age. Women in advanced reproductive age refer to women aged 35–49 years. Pregnancy occurrences among women in this group have been described as ‘advanced maternal age’ [13], but irrespective of pregnancy occurrence or non-occurrence, studies have inadequately investigated factors influencing contraceptive use and non-use among women of advanced reproductive age.

For instance, in two recent studies in Nigeria and Tanzania, a number of factors accounting for childbearing in advanced reproductive age were examined, but there were no specific attention on the dynamics of contraceptive use or non-use among women of advanced reproductive age [14, 15]. Also, a recent assessment of studies focusing on high-risk pregnancies across the world notes the paucity of family planning research on women of advanced reproductive age [16]. Though, a recent Nigerian study focused on contraceptive use among women of advanced reproductive age, however, household decision-making power was the sole explanatory variable examined, and non-use was not investigated in the study [17].

This study thus addresses limitation of previous studies by examining a wider range of factors influencing contraceptive use and non-use among women of advanced reproductive age in Nigeria. It is important to focus on Nigeria for two reasons. Firstly, contraceptive prevalence rate is low in the country with high unmet need for family planning among sexually active women [18]. This might affect the contraceptive behaviour of women of advanced reproductive age. Secondly, being the most populous country in Africa, the adverse effects of unintended pregnancy and childbirth in advanced reproductive age may affect more women and children in Nigeria than in other African countries. The specific objective of the study was to examine factors influencing contraceptive use and non-use among women of advanced reproductive age in Nigeria. This was to provide additional information for enhancing the reproductive health of women through raising awareness of preventing unintended pregnancies among women in advanced reproductive age. The study was guided by the research question: what are the socio-demographic characteristics influencing use or non-use of contraceptive among women of advanced reproductive age in Nigeria?

Women in advanced reproductive age deserve demographic and health research attention for a number of reasons. Firstly, pregnancies among them are high-risk pregnancies with higher likelihood of adverse maternal and perinatal outcomes [13, 19–21]. Effective contraceptive use could help prevent pregnancies that are not intended among all categories of women including women in advanced reproductive age. Secondly, not all of them have attained completed fertility. Some may still be without any child, and some may have just started childbearing [22–24]. In addition, some may have deliberately delayed childbearing in pursuits of post-materialist values as predicted by the post-materialist value theory as well as the Second Demographic Transition Theory [5, 25], giving rise to steady increase in the median age at first marriage and median age at first birth as already being observed in Nigeria. For instance, median age at first marriage in Nigeria increased from 17.1 years in 1990 to 18.3 years in 2013, while median age at first birth increased from 19.7 years to 20.2 over the same period [18, 26]. Hence, many of them are still sexually active. Thirdly, with high unmet need for family planning in several developing countries including Nigeria, there may be likelihood of unintended pregnancies among women of advanced reproductive age [27]. With only 9% of women aged 30–49 years being menopausal in Nigeria, the susceptibility to pregnancy remain high among women in advanced reproductive age [18]. Many of these women do not accept female sterilisation as a fertility regulation method [28, 29], and in the event of unintended pregnancies, may result to induced abortion which remain largely illegal in Nigeria [30], and which may further endanger their reproductive health. Fourthly, there are no specific family planning interventions for women of advanced reproductive age in many developing countries [16]. Fifthly, assisted reproductive technology used to ease complications of pregnancy in advanced reproductive age in developed countries are not widely available to women of advanced reproductive age in developing countries [31].

## Methods

### Study setting

The geographic domain of the study is Nigeria, West Africa. The population of Nigeria is currently estimated at 181.8 million persons. Birth rate is high in the country with a total fertility rate of over five children per woman. Death rate is lower resulting in high natural increase. Infant and maternal mortality rates in Nigeria were among the highest in West Africa. Contraceptive prevalence rates either for all methods or for modern methods were less than 16% in the country [32]. However, population and health policies are being implemented to improve population health in the country. The key population policy is the 2004 National Policy on Population for Sustainable Development. The policy seeks to improve quality of life in the country through expansion of access to reproductive health services, improving safe motherhood programmes, increasing modern contraceptive prevalence by at least 2% yearly, promoting women empowerment and male involvement in reproductive health among other objectives and targets [33]. The policy also seeks to promote the detection, prevention and management of high-risk pregnancies and births. However, research has provided evidence that the policy has not been adequately implemented [34]. This has further aggravated the reproductive health of women in the country. Though, the policy has some specific programmes for some special population groups such as adolescents, refugees and internally displaced persons, the elderly and persons with disabilities, there are no specific programme for women of advanced reproductive age.

### Data source and study design

Data analysed in the study were pooled and extracted from the 2008 and 2013 Nigeria Demographic and Health Survey (NDHS). The essence of the pooling was to improve the reliability and statistical power of the analyses. Samples covered in the surveys followed Demographic and Health Survey (DHS) international survey methodology of selecting samples through two-staged sampling process [35]. All survey staffs were well trained for the purpose of each round of the survey. Eligible men and women included in the surveys were men and women who were permanent residents or visitors in households that were randomly selected. Included visitors must have stayed in the household at least a night preceding the survey. Response rates in the surveys were of comparable international standard with 98% among women interviewed in the 2013 survey. Informed consent preceded all the interviews [18, 36]. The request to access and analyse the dataset was processed formally through online submission of abstract detailing the objective and methodology of the study to MEASURE DHS. Authorisation was granted without delay. Women in advanced reproductive age who were not sexually active and women who were less than 35 years were excluded from analysis. The weighted sample size analysed in the study was 14,450 women.

### Outcome variable

The outcome variable was current contraceptive use which has three possible outcomes, namely non-use (1), using traditional method (2), and using modern method (3). The outcomes of interest were non-use and using modern method. All women who reported non-use of any method were grouped as ‘non-use’ while women who reported using any modern method such as condom, implants, injectables, sterilisation and foaming tablets were grouped as ‘using modern method’. Women who reported use of traditional method such as abstinence, withdrawal and lactational amenorrhea were grouped as ‘using traditional method’.

### Explanatory and control variables

The explanatory variables were a set of socio-demographic characteristics. The demographic characteristics selected for analysis were age, parity, child mortality experience, age at first birth, fertility desire and ideal family size. The selected socio-economic characteristics were maternal education, household wealth, place of residence, employment status, media exposure and geographic region. Three variables, namely remarriage, paternal education and women’s autonomy were selected for statistical control. The selection of the variables was guided by literature [8, 9, 12, 37, 38]. Some of the variables were however re-classified. Parity was classified into three, namely low (two or fewer children ever born), multiparity (three to four children ever born) and grand multiparity (five or more children ever born). Ideal family size was categorised into two, namely small (four or less) and large (five or more). Exposure to mass media was derived from the frequencies of reading newspapers, listening to radio and watching television within a week. Women who reported no frequency of exposure were grouped as ‘none’, women who accessed at least one of the three outlets less than once a week were grouped as ‘low’ while women who accessed all media outlets more than once a week were grouped as ‘moderate’. Two control variables, namely women autonomy and partner education, were included based on their significance in earlier studies [8, 17, 39]. Women autonomy was derived from responses on women’s participation in three household decisions, namely decisions on own health, purchase of large household items and visit to friends and relatives. Women who either took the three decisions solely or takes at least one of the decisions jointly with male partner were grouped as having ‘autonomy’ while women whose male partner or someone else had final say on the decisions were grouped as having ‘no autonomy’. Remarriage was included as a control variable because in Nigeria, remarriage exerts pressure on women to have additional child as a way of consolidating the new union.

### Statistical analyses

All analyses in the study were performed using Stata version 12. Sample socio-demographic characteristics were described using frequency distribution and percentage. Simple cross tabulation was performed to obtain percentage of use and non-use of contraceptives among the respondents. The multinomial logistic regression was applied for two purposes. Firstly, unadjusted multinomial logistic regression coefficients were applied to examine the separate bivariate relationship between use and non-use of contraceptive and the explanatory variables. Secondly, the relative risk ratios (rrr) were applied to examine the multivariate influence of the selected socio-demographic variables on use and non-use of contraceptives.

### Description of the model

The dependent variable being current contraceptive use has three possible outcomes, namely non-use, using traditional method and using modern method. These outcomes are unordered, coded 1, 2 and 3 respectively, and recoded in *y* notation. The explanatory variables are recorded in *X* notation. Three coefficients corresponding to each of the possible outcome of the dependent variable, that is (*β* ^(1)^, *β* ^(2)^, *β* ^(3)^), are to be estimated. The mathematical expression for estimating the coefficients are as follows:

$$ \Pr \left(y=1\right)=\frac{e^{X\beta (1)}}{e^{X\beta (1)+}{e}^{X\beta (2)}+{e}^{X\beta (3)}} $$

$$ \Pr \left(y=2\right)=\frac{e^{X\beta (2)}}{e^{X\beta (1)+}{e}^{X\beta (2)}+{e}^{X\beta (3)}} $$

$$ \Pr \left(y=3\right)=\frac{e^{X\beta (3)}}{e^{X\beta (1)+}{e}^{X\beta (2)}+{e}^{X\beta (3)}} $$

The expression will however be unidentified because it will result in the same probabilities for each of the three possible outcomes. To make the expression identifiable, outcome 2 (using traditional method) was selected as the base outcome. By this selection, change in outcome 1 (non-use) and outcome 3 (using modern method) will be measured relatively to outcome 2. The expression was thus modified as:

$$ \Pr \left(y=1\right)=\frac{e^{X\beta (1)}}{1+{e}^{X\beta (2)}+{e}^{X\beta (3)}} $$

$$ \Pr \left(y=2\right)=\frac{1}{1+{e}^{X\beta (2)}+{e}^{X\beta (3)}} $$

$$ \Pr \left(y=3\right)=\frac{e^{X\beta (3)}}{1+{e}^{X\beta (2)}+{e}^{X\beta (3)}} $$

[36].

### Fitting the model

The multinomial logistic regression model was fitted using the Stata *mlogit* command [40]. The logistic regressions were estimated using the relative risk ratio (rrr). The rrr measures the change in outcome 1 and outcome 3 in relation to the base outcome (2) and was derived from the relative probability of each outcome to the base outcome, that is:

$$ \frac{ \Pr \left(y=1\right)}{ \Pr \left(y=2\right)}={e}^{X{\beta}^{(1)}} $$ and $$ \frac{ \Pr \left(y=3\right)}{ \Pr \left(y=2\right)}={e}^{X{\beta}^{(3)}} $$.

The multinomial logistic regression was replicated in four models. Model 1 was based solely on the demographic variables, while model 2 was based solely on the socio-economic variables. In model 3, the demographic and socio-economic variables were combined. Model 4 was the full model which included all variables including the control variables.

### Model diagnosis

The goodness-of-fit of the model was determined by the likelihood ratio chi-square. The importance of this statistic was to show whether the model fits significantly than an empty model, which is a model not including any of the explanatory variables of the study. Statistical significance was set at 5% (*p* < 0.05). The variance inflation factor (VIF) was performed to detect multicollinearity between the explanatory variables. The mean VIF score of 3.12 confirms the non-existence of serious multicollinearity.

## Results and discussion

Table 1 presents the socio-demographic characteristics of respondents. Majority of the respondents were in the early advanced reproductive age (35–39 years). However, more than half of the women were in older advanced age groups. Majority of the respondents were grand multiparous women. Notwithstanding, slightly more than one tenth of them reported low parity. Though the proportion of respondents who had ever experienced child mortality was higher than the proportion who never experienced child mortality, the proportions were however similar. Majority of the respondents initiated childbearing at age group 15–19 years. However, more than a quarter of the women also became mothers between age 20 and 14 years. Likewise, nearly one fifth of the respondents had their first birth at age 25 years or older. The proportion of respondents who became mothers before reaching the lower limit of the reproductive life span was slightly more than one tenth. More than half of the women do not want another child, while a substantial proportion of them want more children. Majority of the respondents had large ideal family size.

**Table 1:** Socio-demographic characteristics of women of advanced maternal age, Nigeria, 2008 and 2013

Characteristic	Number of women (*n* = 14,450)	Percentage (100.0)
Maternal age		
35–39 years	6110	42.3
40–44 years	4554	31.5
45–49 years	3786	26.2
Parity		
Low	1575	10.9
Multiparity	2847	19.7
Grand multiparity	10,028	69.4
Child mortality experience		
Ever experienced	7456	51.6
Never experienced	6994	48.4
Age at first birth		
14 years or less	1789	12.4
15–19 years	5978	41.4
20–24 years	4039	27.9
25 years or older	2644	18.3
Fertility desire		
Wants no more children	7898	54.7
Wants more children	6464	44.7
Undecided	88	0.6
Ideal family size		
Small	2644	18.3
Large	11,806	81.7
Maternal education		
None	7305	50.6
Primary	3038	21.0
Secondary	2808	19.4
Higher	1299	9.0
Household wealth index		
Poorest	3475	24.1
Poorer	2817	19.5
Middle	2463	17.0
Richer	2546	17.6
Richest	3149	21.8
Place of residence		
Urban	5311	36.8
Rural	9139	63.2
Employment status		
Not working	3248	22.5
Working	11,202	77.5
Media exposure		
None	4269	29.6
Low	2705	18.7
Moderate	7476	51.7
Geographic region		
Northern region	9083	62.9
Southern region	5367	37.1
Current contraceptive use		
Non-use	11,582	80.2
Using traditional method	1048	7.2
Using modern method	1820	12.6
Remarriage		
Married once	11,778	81.5
Remarried	2672	18.5
Women autonomy		
No autonomy	6246	43.2
Autonomy	8204	56.8

Source: Author analysis based on 2008 and 2013 Nigeria Demographic and Health Survey

More than half of respondents had no formal education. Among respondents with educational attainments, primary education was dominant. Majority of the respondents were in the lowest wealth groups with nearly a quarter in the ‘poorest’ wealth group. Though more than one third of the respondents reside in urban areas, majority of the respondents reside in rural areas. Regardless of occupational category, majority of the respondents were employed. More than half of the respondents had moderate access to mass media outlets. Respondents from the northern region of the country were dominant in the sample. Majority of the respondents are not currently using any method of contraceptive. Among current users, slightly more than one tenth are using modern method, while less than one tenth of them are using traditional method. Majority of the respondents have been married only once; however, nearly one-fifth of respondents had remarried. More than half of the women had individual autonomy on household decisions. However, a substantial proportion of them do not have individual autonomy on household decisions. Nearly half of the respondents’ partners had no formal education. Among partners with educational attainment, secondary education was dominant (data not shown in Table 1).

Table 2 presents the bivariate relationship between socio-demographic characteristics and non-use of contraceptive. Higher proportions of non-users were at the extreme interval of the reproductive life span. At 40–44 years, maternal age was negatively associated with non-use of contraceptive, but at age 45–49 years, the relationship was positive. With exclusion of women with low parity, proportions of non-users increased with parity level. Grand multiparous women had higher proportion of non-users of contraceptive. The relationship between parity and non-use of contraceptive was negative. Child mortality experience was negatively associated with non-use of contraceptive with higher proportion of non-users among women who had ever experienced child mortality. Likewise, age at first birth was negatively associated with non-use of contraceptive with declining proportions of non-users as age at first birth increases. Fertility desire relates positively with non-use of contraceptive with higher proportion of non-users among women who desire more children compared with women who do not want additional child (71.9% vs. 90.4%). Ideal family size and non-use of contraceptive were positively related with higher proportion of non-users among women who had large ideal family size compared with women who had small ideal family size (60.8% vs. 84.5%).

**Table 2:** Proportion not using any method and unadjusted regression coefficient showing bivariate relationship

Characteristic	Non-use (%)	Coefficient	*p* > |*z*|	95% CI
Maternal age				
35–39 years (ref)	77.7	–	–	–
40–44 years	77.7	−0.054	0.471	−0.201 to −0.093
45–49 years	87.1	0.692	<0.001	0.503 to 0.880
Parity				
Low parity (ref)	89.6	–	–	–
Multiparity	66.8	−1.373	<0.001	−1.666 to −1.080
Grand multiparity	82.4	−0.506	<0.001	−0.787 to −0.226
Child mortality experience				
Ever experienced (ref)	87.1	–	–	–
Never experienced	72.7	−0.927	<0.001	−1.067 to −0.788
Age at first birth				
14 years or less (ref)	91.4	–	–	–
15–19 years	84.5	−0.721	<0.001	−1.047 to −0.396
20–24 years	73.6	−1.457	<0.001	−1.780 to −1.135
25 years or older	72.7	−1.736	<0.001	−2.064 to −1.408
Fertility desire				
Wants no more children (ref)	71.9	–	–	–
Want more children	90.4	1.029	<0.001	0.878 to 1.179
Undecided	64.6	−0.173	0.630	−0.875 to 0.529
Ideal family size				
Small (ref)	60.8	–	–	–
Large	84.5	1.213	<0.001	1.069 to 1.357
Maternal education				
None (ref)	95.5	–	–	–
Primary	72.9	−1.785	<0.001	−2.004 to −1.566
Secondary	58.9	−2.514	<0.001	−2.723 to −2.304
Higher	56.8	−2.748	<0.001	−2.983 to −2.514
Household wealth index				
Poorest (ref)	97.3	–	–	–
Poorer	91.9	−0.876	<0.001	−1.257 to −0.494
Middle	82.4	−1.809	<0.001	−2.158 to −1.460
Richer	70.7	−2.490	<0.001	−2.825 to −2.156
Richest	56.7	−3.066	<0.001	−3.395 to −2.742
Place of residence				
Urban (ref)	67.0	–	–	–
Rural	87.8	1.246	<0.001	1.110 to 1.381
Employment status				
Not working (ref)	92.6	–	–	–
Working	76.6	−1.393	<0.001	−1.632 to −1.153
Media exposure				
None (ref)	93.5	–	–	–
Low	81.8	−1.258	<0.001	−1.515 to −1.000
Moderate	71.9	−1.786	<0.001	−2.005 to −1.568
Geographic region				
Northern region (ref)	91.9	–	–	–
Southern region	60.3	−2.550	<0.001	−2.723 to −2.378

*p* value greater than 0.05 indicate not significant

*ref* reference category

Maternal education was negatively related with non-use of contraceptive. As educational level improves, the proportions of non-users reduce consistently. Likewise, the proportions of non-users reduce consistently as household wealth status improves showing negative relationship between household wealth and non-use of contraceptive. Place of residence and non-use of contraceptive were positively associated with higher proportion of non-users among rural women. Employment status was negatively related with non-use of contraceptive with higher proportion of non-users among women who were not working compared with those working (92.6% vs. 76.6%). Likewise, media exposure and non-use of contraceptive were negatively related. As exposure to mass media improves, the proportion of non-users reduces. The relationship between geographic region and non-use of contraceptive was negative with higher proportion of non-users in northern Nigeria compared with southern Nigeria (91.9% vs. 60.3%).

Table 3 presents the bivariate relationship between socio-demographic characteristics and modern contraceptive use. The proportions of women using modern contraceptive were higher among younger women compared with older women. Thus, the relationship between maternal age and modern contraceptive use was negative at younger ages, but positive at the older age group. With exclusion of women with low parity, proportions of women using modern contraceptive reduces with parity level. Grand multiparous women had lower proportion of modern contraceptive use compared with multiparous women (11.4% vs. 20.1%). The relationship between parity and modern contraceptive use was negative among multiparous women, but positive among grand multiparous women. Child mortality experience was negatively associated with modern contraceptive use with higher proportion of modern contraceptive use among women who had never experienced child mortality compared with women who had ever experienced child mortality (17.3% vs. 8.2%). The proportions of women using modern contraceptive was mixed among the women. However, the age at first birth was negatively associated with modern contraceptive use irrespective of age at initiating childbearing. Fertility desire relates negatively with modern contraceptive use with higher proportion of women using modern contraceptive among women who do not want more children compared with women who want more children (18.5% vs. 5.1%). Ideal family size and modern contraceptive use were positively related with higher proportion of women using modern contraceptive among women who had small ideal family size compared with women who had large ideal family size (24.6% vs. 9.9%).

**Table 3:** Proportion using modern contraceptive and unadjusted regression coefficient showing bivariate relationship

Characteristic	Non-use (%)	Coefficient	*p* > |*z*|	95% CI
Maternal age				
35–39 years (ref)	14.1	–	–	–
40–44 years	13.9	−0.068	0.444	−0.243 to 0.106
45–49 years	8.6	0.121	0.290	−0.103 to 0.344
Parity				
Low parity (ref)	6.3	–	–	–
Multiparity	20.1	−0.090	0.616	−0.444 to 0.263
Grand multiparity	11.4	0.055	0.751	−0.284 to 0.394
Child mortality experience				
Ever experienced (ref)	8.2	–	–	–
Never experienced	17.3	−0.031	0.717	−0.197 to 0.135
Age at first birth				
14 years or less (ref)	6.1	–	–	–
15−19 years	10.3	−0.135	0.487	−0.514 to 0.245
20−24 years	17.2	−0.280	0.144	−0.657 to 0.096
25 years or older	15.1	−0.581	0.003	−0.966 to −0.196
Fertility desire				
Wants no more children (ref)	18.5	–	–	–
Want more children	5.1	−0.416	<0.001	−0.604 to −0.227
Undecided	27.4	0.257	0.514	−0.514 to 1.028
Ideal family size				
Small (ref)	24.6	–	–	–
Large	9.9	0.014	0.869	−0.152 to 0.179
Maternal education				
None (ref)	2.7	–	–	–
Primary	18.4	0.266	0.049	0.008 to 0.532
Secondary	26.0	0.066	0.608	−0.188 to 0.321
Higher	25.5	−0.008	0.955	−0.288 to 0.271
Household wealth index				
Poorest (ref)	1.4	—	–	–
Poorer	5.3	0.298	0.220	−0.178 to 0.774
Middle	11.9	0.254	0.254	−0.183 to 0.691
Richer	18.2	0.182	0.396	−0.238 to 0.602
Richest	27.6	0.164	0.431	−0.245 to 0.574
Place of residence				
Urban (ref)	20.9	–	–	–
Rural	7.8	0.046	0.568	−0.113 to 0.206
Employment status				
Not working (ref)	4.9	–	–	–
Working	14.8	−0.181	0.212	−0.466 to 0.103
Media exposure				
None (ref)	4.2	–	–	–
Low	11.2	−0.117	0.459	−0.428 to 0.193
Moderate	17.9	−0.053	0.695	−0.316 to 0.210
Geographic region				
Northern region (ref)	6.6	–	–	–
Southern region	22.8	−1.100	<0.001	−1.293 to −0.907

*p* value greater than 0.05 indicate not significant

*ref* reference category

Maternal education had mixed relationship with modern contraceptive use. At lower educational levels, the relationship was positive, but at higher educational level, the relationship was negative. The proportions of women using modern contraceptive increase consistently as household wealth status improves showing positive relationship between household wealth and modern contraceptive use. Place of residence and modern contraceptive use were positively associated with higher proportion of women using modern contraceptive among urban than rural women (20.9% vs. 7.8%). Employment status was negatively associated with modern contraceptive use with higher proportion of women using modern contraceptive among women who were working compared with those not working (14.8% vs. 4.9%). Media exposure and modern contraceptive use were negatively related. As exposure to mass media improve, the proportion of women using modern contraceptive increases. The relationship between geographic region and modern contraceptive use was negative with higher proportion of women using modern contraceptive in southern than northern Nigeria (22.8% vs. 6.6%).

Table 4 presents the result of multivariate influence on non-use of contraceptive. Model information shown in the table confirm goodness-of-fit of the models. In model 1, all demographic characteristics analysed significantly influence non-use of contraceptive. Likewise, in model 2, all socio-economic characteristics analysed exerts significant influence on the likelihood of not using contraceptive. With the combination of both socio-economic and demographic characteristics in model 3, there were minimal changes in the pattern of influence on non-use of contraceptive. All the socio-demographic characteristics remain significant predictors of non-use of contraceptive. However, there was marked variation in the pattern of influence shown in the full model. The expected risk of not using contraceptive relative to traditional method was higher for women aged 45–49 years compared with women aged 35–39 years (rrr = 2.694; CI: 2.174–3.340). For a unit change in parity, the expected risk of being in the non-user category relative to traditional method user category decreases by a factor of 0.286 among multiparous women (rrr = 0.286; CI: 0.207–0.396) while it decreases by a factor of 0.193 among grand multiparous women (rrr = 0.193; CI: 0.134–0.277).

**Table 4:** Relative risk ratios showing influence on non-use of contraceptive with traditional method as base outcome

Characteristic predicting non-use of contraceptive	Model 1 (demographic) LR *χ* ^2^ (22) = 2283.0; *p* < 0.001			Model 2 (socio-economic) LR *χ* ^2^ (24) = 3148.4; *p* < 0.001			Model 3 (socio-demographic) LR *χ* ^2^ (46) = 4285.8; *p* < 0.001			Model 4 (full) LR *χ* ^2^ (58) 4730.8; *p* < 0.001		
	RRR	*p* > |*z*|	95% CI	RRR	*p* > |*z*|	95% CI	RRR	*p* > |*z*|	95% CI	RRR	*p* > |*z*|	95% CI
Maternal age												
35–39 years (ref)	–	–	–				–	–	–	–	–	–
40–44 years	1.146	0.086	0.981 to 1.340				1.105	0.236	0.937 to 1.303	1.100	0.259	0.932 to 1.230
45–49 years	2.914	<0.001	2.380 to 3.568				2.710	<0.001	2.191 to 3.351	2.694	<0.001	2.174 to 3.340
Parity												
Low (ref)	–	–	–				–	–	–	–	–	–
Multiparity	0.324	<0.001	0.238 to 0.442				0.272	<0.001	0.197 to 0.376	0.286	<0.001	0.207 to 0.396
Grand multiparity	0.312	<0.001	0.225 to 0.433				0.179	<0.001	0.125 to 0.255	0.193	<0.001	0.135 to 0.277
Child mortality experience												
Ever experienced (ref)	–	–	–				–	–	–	–	–	–
Never experienced	0.499	<0.001	0.427 to 0.583				0.676	<0.001	0.574 to 0.796	0.688	<0.001	0.583 to 0.811
Age at first birth												
14 years or less (ref)	–	–	–				–	–	–	–	–	–
15–19 years	0.538	<0.001	0.386 to 0.750				0.661	0.018	0.469 to 0.932	0.704	0.046	0.498 to 0.994
20–24 years	0.277	<0.001	0.199 to 0.386				0.480	<0.001	0.340 to 0.677	0.513	<0.001	0.363 to 0.725
25 years or older	0.188	<0.001	0.134 to 0.266				0.368	<0.001	0.257 to 0.527	0.399	<0.001	0.278 to 0.572
Fertility desire												
Wants more children (ref)	–	–	–				–	–	–	–	–	–
Want no more children	3.336	<0.001	2.820 to 3.946				2.470	<0.001	2.059 to 2.962	2.481	<0.001	2.064 to 2.982
Undecided	1.039	0.918	0.501 to 2.153				0.604	0.199	0.280 to 1.302	0.560	0.144	0.257 to 1.219
Ideal family size												
Small (ref)	–	–	–				–	–	–	–	–	–
Large	2.456	<0.001	2.073 to 2.910				1.488	<0.001	1.236 to 1.791	1.480	<0.001	1.228 to 1.785
Maternal education												
None (ref)				–	–	–	–	–	–	–	–	–
Primary				0.579	<0.001	0.449 to 0.746	0.662	0.002	0.513 to 0.856	0.891	0.403	0.679 to 1.168
Secondary				0.433	<0.001	0.332 to 0.565	0.516	<0.001	0.394 to 0.677	0.687	0.011	0.515 to 0.918
Higher				0.344	<0.001	0.255 to 0.462	0.355	<0.001	0.260 to 0.485	0.475	<0.001	0.336 to 0.671
Place of residence												
Urban (ref)				–	–	–	–	–	–	–	–	–
Rural				1.332	0.001	1.129 to 1.571	1.342	0.001	1.130 to 1.593	1.333	0.001	1.121 to 1.584
Household wealth index												
Poorest (ref)				–	–	–	–	–	–	–	–	–
Poorer				0.773	0.202	0.521 to 1.148	0.845	0.410	0.567 to 1.261	1.018	0.931	0.679 to 1.527
Middle				0.596	0.008	0.407 to 0.872	0.636	0.021	0.433 to 0.934	0.841	0.387	0.569 to 1.244
Richer				0.534	0.002	0.361 to 0.789	0.574	0.006	0.387 to 0.853	0.763	0.188	0.511 to 1.141
Richest				0.460	<0.001	0.305 to 0.693	0.510	0.002	0.336 to 0.776	0.695	0.096	0.453 to 1.067
Employment status												
Not working (ref)				–	–	–	–	–	–	–	–	–
Working				0.626	<0.001	0.485 to 0.808	0.632	0.001	0.487 to 0.821	0.716	0.013	0.550 to 0.932
Media exposure												
None (ref)				–	–	–	–	–	–	–	–	–
Low				0.786	0.093	0.594 to 1.041	0.757	0.056	0.569 to 1.007	0.869	0.336	0.652 to 1.157
Moderate				0.722	0.012	0.561 to 0.931	0.710	0.009	0.549 to 0.920	0.814	0.120	0.628 to 1.055
Geographic region												
Northern (ref)				–	–	–	–	–	–	–	–	–
Southern				0.190	<0.001	0.155 to 0.232	0.195	<0.001	0.159 to 0.239	0.223	<0.001	0.181 to 0.275
Remarriage												
Married once (ref)										–	–	–
Remarried										1.355	0.014	1.064 to 1.727
Paternal education												
None (ref)										–	–	–
Primary										0.648	0.003	0.487 to 0.862
Secondary										0.774	0.091	0.575 to 1.042
Higher										0.787	0.154	0.566 to 1.094
Women autonomy												
No autonomy										–	–	–
Autonomy										0.844	0.082	0.697 to 1.022

*p* value greater than 0.05 indicate not significant

*ref* reference category

As age at first birth increases, the expected risk of being a non-user of contraceptive relative to being a traditional method user reduces consistently. For instance, for women who had their first live birth at 25 years or older, the relative risk for not using contraceptive relative to using traditional method reduces by a factor of 0.399 (rrr = 0.399; CI: 0.278–0.572) compared with women in the reference category of 14 years or younger. Likewise, the relative risk for not using contraceptive relative to using traditional method was lower among women who had never experienced child mortality compared with women who had experienced child mortality (rrr = 0.688; CI: 0.583–0.811). The expected risk of not using contraceptive relative to using traditional method increase by a factor of 2.481 for women who want more children compared with women who no longer had fertility desire (rrr = 2.481; CI: 2.064–2.982). Similarly, the expected risk of not using contraceptive relative to using traditional method increase by a factor of 1.480 for women who had large ideal family size compared with women who had small ideal family size (rrr = 1.480; CI: 1.228–1.785).

As maternal education improve, the expected risk of not using contraceptive relative to using traditional method reduces but with no statistical significance at primary education. However, at higher education, the expected risk of not using contraceptive relative to traditional method reduces by a factor of 0.475 (rrr = 0.475; CI: 0.336–0.671). Household wealth did not reveal significant influence on non-use of contraceptive. The expected risk of not using contraceptive relative to using traditional method increase by a factor of 1.333 among rural women compared with urban women (rrr = 1.333; CI: 1.121–1.584). The relative risk for not using contraceptive relative to using traditional method was lower for women who were working compared with women not working (rrr = 0.716; CI: 0.550–0.932). Media exposure, paternal education and women autonomy did not impact non-use of contraceptive. The expected risk of not using contraceptive relative to using traditional method was lower for southern women compared with northern women (rrr = 0.223; CI: 0.181–0.275). The expected risk of not using contraceptive relative to using traditional method increase by a factor of 1.355 among women who remarried compared with women who married only once (rrr = 1.355; CI: 1.064–1.727).

Table 5 presents the result of multivariate influence on modern contraceptive use. Model information shown in the table confirm goodness-of-fit of the models. In model 1, only fertility desire significantly influences modern contraceptive use. However, age at first birth exert impact on modern contraceptive use among women who had their first live birth at age 25 years or older. In model 2, maternal education, household wealth and geographic region were the socio-economic characteristics that reveal significant influence on modern contraceptive use. When both socio-economic and demographic characteristics were combined in model 3, parity and age at first birth had mixed effects on modern contraceptive use, while fertility desire, maternal education, household wealth and geographic region maintained significant influence on modern contraceptive use. In the full model, four demographic characteristics, namely maternal age, child mortality experience, age at first birth and ideal family size did not reveal significant influence on modern contraceptive use, while two demographic characteristics, namely parity and fertility desire significantly influence modern contraceptive use. The relative risk for using modern contraceptive relative to traditional method reduces by a factor of 0.676 for multiparous women (rrr = 0.676; CI: 0.464–0.985). Likewise, the relative risk for using modern contraceptive relative to traditional method reduces by a factor of 0.622 for grand multiparous women (rrr = 0.622; CI: 0.413–0.938). The expected risk of using modern contraceptive relative to traditional method reduces by a factor of 0.611 for women who want more children compared with women who do not want more children (rrr = 0.611; CI: 0.493–0.757).

**Table 5:** Relative risk ratios showing influence on modern contraceptive use with traditional method as base outcome

Characteristic predicting modern contraceptive use	Model 1 (demographic) LR *χ* ^2^ (22) = 2283.0; *p* < 0.001			Model 2 (socio-economic) LR *χ* ^2^ (24) =3148.4; *p* < 0.001			Model 3 (socio-demographic) LR *χ* ^2^ (46) = 4285.8; *p* < 0.001			Model 4 (full) LR *χ* ^2^ (58) 4730.8; *p* < 0.001		
	RRR	*p* > |z|	95% CI	RRR	*p* > |*z*|	95% CI	RRR	*p* > |*z*|	95% CI	RRR	*p* > |*z*|	95% CI
Maternal age												
35–39 years (ref)	–	–	–				–	–	–	–	–	–
40–44 years	0.891	0.204	0.745 to 1.065				0.895	0.231	0.747 to 1.073	0.883	0.182	0.736 to 1.060
45–49 years	1.061	0.618	0.841 to 1.338				1.117	0.355	0.883 to 1.413	1.107	0.402	0.873 to 1.403
Parity												
Low (ref)	–	–	–				–	–	–	–	–	–
Multiparity	0.800	0.230	0.555 to 1.152				0.691	0.053	0.475 to 1.005	0.676	0.042	0.464 to 0.985
Grand multiparity	0.791	0.229	0.539 to 1.159				0.629	0.026	0.419 to 0.946	0.622	0.024	0.413 to 0.938
Child mortality experience												
Ever experienced (ref)	–	–	–				–	–	–	–	–	–
Never experienced	1.028	0.766	0.858 1.232				1.014	0.878	0.844 1.218	1.030	0.751	0.857 1.238
Age at first birth												
14 years or less (ref)	–	–	–				–	–	–	–	–	–
15–19 years	0.894	0.566	0.609 to 1.311				0.915	0.654	0.621 to 1.349	0.917	0.664	0.621 to 1.353
20–24 years	0.776	0.192	0.530 to 1.136				0.855	0.429	0.580 to 1.260	0.860	0.449	0.583 to 1.270
25 years or older	0.575	0.006	0.386 to 0.856				0.663	0.047	0.442 to 0.993	0.668	0.052	0.444 to 1.004
Fertility desire												
Wants more children (ref)	–	–	–				–	–	–	–	–	–
Want no more children	0.677	<0.001	0.552 to 0.830				0.619	<0.001	0.500 to 0.766	0.611	<0.001	0.493 to 0.757
Undecided	1.367	0.428	0.631 to 2.965				1.053	0.898	0.477 to 2.326	1.061	0.884	0.479 to 2.348
Ideal family size												
Small (ref)	–	–	–				–	–	–	–	–	–
Large	0.989	0.909	0.818 to 1.196				0.937	0.523	0.767 to 1.144	0.948	0.603	0.775 to 1.159
Maternal education												
None (ref)				–	–	–	–	–	–	–	–	–
Primary				2.321	<0.001	1.718 to 3.137	2.232	<0.001	1.652 to 3.015	1.916	<0.001	1.397 to 2.626
Secondary				2.032	<0.001	1.483 to 2.783	1.982	<0.001	1.445 to 2.718	1.637	0.004	1.173 to 2.285
Higher				1.610	0.007	1.138 to 2.280	1.587	0.011	1.112 to 2.263	1.192	0.377	0.807 to 1.759
Place of residence												
Urban (ref)				–	–	–	–	–	–	–	–	–
Rural				1.048	0.623	0.869 to 1.264	1.032	0.748	0.853 to 1.248	1.018	0.856	0.840 to 1.233
Household wealth index												
Poorest (ref)				–	–	–	–	–	–	–	–	–
Poorer				1.580	0.066	0.971 to 2.572	1.613	0.055	0.989 to 2.631	1.550	0.081	0.947 to 2.534
Middle				1.823	0.012	1.139 to 2.916	1.771	0.017	1.106 to 2.834	1.632	0.043	1.016 to 2.622
Richer				1.973	0.005	1.222 to 3.185	1.883	0.010	1.166 to 3.043	1.722	0.027	1.062 to 2.791
Richest				2.110	0.003	1.280 to 3.476	1.954	0.009	1.183 to 3.229	1.726	0.035	1.038 to 2.871
Employment status												
Not working (ref)				–	–	–	–	–	–	–	–	–
Working				1.036	0.812	0.775 to 1.389	1.020	0.897	0.759 to 1.370	0.967	0.823	0.718 to 1.300
Media exposure												
None (ref)				–	–	–	–	–	–	–	–	–
Low				0.912	0.583	0.656 to 1.267	0.937	0.699	0.673 to 1.304	0.897	0.519	0.645 to 1.248
Moderate				0.973	0.860	0.723 to 1.311	0.986	0.929	0.731 to 1.331	0.936	0.663	0.694 to 1.261
Geographic region												
Northern (ref)				–	–	–	–	–	–	–	–	–
Southern				1.237	<0.001	1.190 to 1.805	1.241	<0.001	1.193 to 1.781	1.250	<0.001	1.200 to 1.818
Remarriage												
Married once (ref)										–	–	–
Remarried										1.246	0.112	0.950 to 1.635
Paternal education												
None (ref)										–	–	–
Primary										0.999	0.994	0.718 to 1.389
Secondary										1.023	0.896	0.728 to 1.436
Higher										1.281	0.189	0.885 to 1.854
Women autonomy												
No autonomy (ref)										–	–	–
Autonomy										1.201	0.098	0.967 to 1.492

*p* value greater than 0.05 indicate not significant

*ref* reference category

Only three socio-economic characteristics exert significant influence on modern contraceptive use. As maternal education improves from none to primary education, the expected risk of using modern contraceptive relative to traditional method increases by a factor of 1.916 (rrr = 1.916; CI: 1.397–2.626) and by a factor of 1.637 as maternal education reaches secondary education (rrr = 1.637; CI: 1.173–2.285). However, at higher education, the higher relative risk for using modern contraceptive was without statistical significance. As household wealth improves, the expected risk of using modern contraceptive relative to traditional method increases consistently. For instance, for women in richer households, the relative risk for using modern contraceptive relative to traditional method increases by a factor of 1.722 (rrr = 1.722; CI: 1.062–2.791) for women in richer households and by a factor of 1.726 for women in richest households (rrr = 1.726; CI: 1.038–2.871). The expected risk of using modern contraceptive relative to traditional method increases by a factor of 1.250 for southern women compared with northern women (rrr = 1.250; CI: 1.200–1.818).

This study has dual strength. Firstly, the study contributed to knowledge by providing information about dynamics of contraceptive use and non-use among women of advanced reproductive age which represent a group of women less researched in relation to contraceptive behaviour. Most existing studies on women of advanced reproductive age have often pay attention to adverse maternal and perinatal outcomes [13, 20–22] without raising question about what influences or hinders contraceptive use among the women. If women in advanced reproductive age use contraceptives effectively particularly modern contraceptives, concerns about adverse maternal and perinatal outcomes among them will reduce substantially because rate of unintended pregnancies is also likely to reduce among them. Secondly, the nature and quality of the data analysed constitute major strength of the study. On the one hand, the pooling of 2008 and 2013 NDHS data enhances the precision of the analysis. On the other hand, the data analysed were of high quality and comparable to data collected in comparable nationally representative samples. Three key issues emerged from the analyses.

Firstly, the study confirms that contraceptive prevalence among women of advanced reproductive age in the country is at the same low level usually found among younger or other groups of women in the country. Though, the 12.6% prevalence of modern contraceptive found in the study was slightly lower than the 18.7% reported in a recent study [17] for similar group of women, the variation was permissible due to differences in the rounds of NDHS analysed in the two studies. The low use of modern contraceptive among women of advanced reproductive age is usually erroneously assumed to be either due to sexual inactivity or completed fertility among women at the extreme end of the reproductive ladder. However, as found in the study, some women though at advanced reproductive age are low-parity women. It is not certain that such women no longer desire additional children. It is also not certain that the multiparous and grand multiparous women among them also do not want more children. This is why further attention on the contraceptive behaviour of women in advanced reproductive age is important in the country.

Since research has provided evidence of higher likelihood of adverse maternal and perinatal outcomes among this group of women, it is more plausible to channel efforts to preventing unintended pregnancies among the women than expanding safe motherhood programmes to address the complications that may arise from high-risk pregnancies among the women. Though, the existing population policy sought to promote measures for detecting, preventing and managing high-risk pregnancies in the country, specific measure focusing on women of advanced reproductive age has not been developed compared with measures developed to address population issues among younger people. For instance, a recent assessment of the existing policy noted that though the policy education target has not been fully achieved, strategies such as Girls Education Programme designed to achieve policy target of eliminating gap between men and women in enrolment in educational institutions have achieved relative success in Northern Nigeria, and National Gender Parity Index for secondary school enrolment has increased from 0.77 in 2003 to 0.86 in 2014 [34]. It is however possible to strengthen some of the existing strategies in the policy to address contraceptive concerns of women of advanced reproductive age. For instance, one of the key strategies of the policy is the promotion of Behaviour Change Communication (BCC). This strategy thrives through dissemination of population and reproductive health messages. The scope, content and coverage of the messages could be extended to cover the contraceptive needs and challenges of women of advanced reproductive age. This, however, should be complemented by raising awareness about demographic issues such as early age at first birth, high fertility norms and high parity and addressing high child mortality which interacts to sustain non-use of contraceptive among women as found in the study. The messages should be more widely disseminated in northern Nigeria where results confirm less likelihood of modern contraceptive use.

Secondly, there is need for renewed commitment to social programmes to address the socio-economic status of women in the country particularly women in advanced reproductive age groups. As found in the study and consistent with finding in a recent study [12], women with lower socio-economic characteristics such as uneducated women, unemployed women and women in poorest household had higher likelihood of not using contraceptive in spite of their advanced age. This not only increases the risk of unintended pregnancies among them but also increases lifetime risk of maternal deaths or disabilities among them. Existing policy initiatives have stressed expanding contraceptive distribution and logistics; it is however doubtful that contraceptive prevalence will improve in the country without corresponding improvement in the socio-economic status of women. Large numbers of women still do not have access to basic education, unemployment is more widespread among women, and several socio-cultural practices such as property inheritance, child marriages, land tenure system and gender-based violence still adversely affect economic productivity of women in the country [33]. Such social condition does not encourage contraceptive use irrespective of age. It is therefore important for government in the country to give more recognition to the fact that social condition of women in the country may hinder the attainment of the contraceptive target of increasing modern contraceptive prevalence by 2% yearly. An avenue for demonstrating such recognition is to ensure that women concerns are integrated into all social and economic development planning programmes at all levels of governance in the country.

Thirdly, the predictors of contraceptive use among women of advanced reproductive age are more complex than the predictors of non-use of contraceptive among the same group of women. Findings from the study reveal that maternal and household socio-demographic characteristics to a large extent influences non-use of contraceptive, but most of these characteristics fail to significantly influence modern contraceptive use. This implies that interventions aiming to improve contraceptive behaviour of women of advanced reproductive age should endeavour to search for contributory factors beyond individual and household levels. In Nigeria and several sub-Saharan African countries, several socio-cultural norms and practices in traditional communities particularly in rural areas impact childbearing and reproductive behaviour of women across all age groups. Such practices include gender preference, and widow inheritance, and may pressurise women to keep having children at advanced ages. Such cultural concerns must be brought to the fore of population policies and programming in the country.

Data analysed in the study are not only cross-sectional but they are also solely quantitative. The implication of this is that no cause-effect relationship has been established by the study. The analyses carried out in the study have however been able to establish significant association between the research variables. The use of only quantitative data in the study limits detail insights into the reasons that accounts for contraceptive use or non-use among the group of women studied. A follow-up study is proposed to adopt a mixed-method approach to further investigate contraceptive behaviour of women at the upper strata of the reproductive life span. It was not practically possible for the model specified in the study to have captured all factors that may either enhance or hinder contraceptive use among the women studied. Future studies on the same theme could be built around other variables not analysed in the study.

## Conclusions

The study examined factors influencing contraceptive use and non-use among women of advanced reproductive age in Nigeria. Data were pooled and extracted from the 2008 and 2013 Nigeria Demographic and Health Surveys. It was evident from the study that maternal age, parity, age at first birth, child mortality experience, fertility desire, ideal family size, maternal education, place of residence, employment status, geographic region and remarriage significantly influenced non-use of contraceptive. However, only a handful of the characteristics, namely parity, fertility desire, maternal education, household wealth and geographic region, significantly influenced modern contraceptive use. These provided answer to the research question. The scope, content and coverage of existing BCC messages should be extended to cover the contraceptive needs and challenges of women of advanced reproductive age in the country.

## Abbreviations

BCC: Behaviour Change Communication
DHS: Demographic and Health Survey
NDHS: Nigeria Demographic and Health Survey
